# Retraction: Modulation of *miR-146a*/complement factor H-mediated inflammatory responses in a rat model of temporal lobe epilepsy

**DOI:** 10.1042/BSR-2016-0290_RET

**Published:** 2024-10-07

**Keywords:** complement factor H, hippocampus, microRNA-146a, neuroinflammation, temporal lobe epilepsy

This article “Modulation of *miR-146a*/complement factor H-mediated inflammatory responses in a rat model of temporal lobe epilepsy” DOI: 10.1042/BSR20160290 is being retracted from *Bioscience Reports* at the request of the Editor-in-Chief and the Editorial Board. This follows receipt of a notification from a reader, alerting the Editorial Board to Figure 4E and Figure 4H, which seem to appear in a previous publication by Yan et al, 2015 (DOI: 10.18632/oncotarget.5012). The authors were contacted regarding the concerns raised. The authors stated there were errors in the data compilation and offered to provide supplementary original data. The authors were asked to provide the original data for Figure 4E and Figure 4H, but did not respond. Given the issues raised, the Editorial Board stand by the decision to retract the article. The authors were contacted regarding the retraction, but did not respond.

